# Cobalt ions induce a cellular senescence secretory phenotype in human synovial fibroblast-like cells that may be an early event in the development of adverse local tissue reactions to hip implants

**DOI:** 10.1016/j.ocarto.2024.100490

**Published:** 2024-05-15

**Authors:** Michael P. Grant, Raheef Alatassi, Mohamad Omar Diab, Mohammed Abushal, Laura M. Epure, Olga L. Huk, Stephane G. Bergeron, Hee-Jeong Im Sampen, John Antoniou, Fackson Mwale

**Affiliations:** a Department of Surgery, McGill University, Montreal, Canada; b SMBD-Jewish General Hospital, Lady Davis Institute for Medical Research, 3755 Cote Ste-Catherine Road, Room F-602, Montreal, Quebec, H3T 1E2, Canada; c Department of Biomedical Engineering, University of Illinois Chicago, IL, USA

**Keywords:** Arthroplasty, Metal ions, Synovial fibroblast, Cobalt, Senescence

## Abstract

**Objectives:**

Total hip arthroplasty is a successful procedure for treating advanced osteoarthritis (OA). Metal bearing surfaces remain one of the most widely implanted prosthesis, however approximately 10% of patients develop adverse local tissue reactions (ALTRs), namely lymphocytic predominant soft tissue reaction with or without necrosis and osteolysis resulting in high revision rates. The mechanism(s) for these reactions remains unclear although T lymphocyte mediated type IV hypersensitivity to cobalt (Co) and chromium (Cr) ions have been described. The purpose of this study was to determine the prolonged effects of Co and Cr metal ions on synovial fibroblasts to better understand the impact of the synovial membrane in the development of ALTRs.

**Methods:**

Human synovial fibroblast-like cells were isolated from donors undergoing arthroplasty. DNA content and Alamar blue assay were used to determine cellular viability against exposure to Co and Cr. A beta-galactosidase assay was used to determine the development of cellular senescence. Western blotting and RT-qPCR were employed to determine changes in senescent associated secretory factors, signaling and anti-oxidant enzyme expression. A fluorescent assay was used to measure accumulation of hydrogen peroxide.

**Results:**

We demonstrate that prolonged cobalt exposure results in a downregulation of the enzyme catalase resulting in cytosolic accumulation of hydrogen peroxide, decreased Akt activity and cellular senescence. Senescent fibroblasts demonstrated upregulation of proinflammatory cytokines IL-1β and TNFα in addition to the neurotrophic factor NGF.

**Conclusion:**

Our results provide evidence that metal ions induce a senescent associated secretory phenotype in synovial fibroblasts that could contribute to the development of adverse local tissue reactions.

## 1 Introduction

Osteoarthritis (OA) is the most common type of arthritis worldwide, affecting approximately 15% of the world's population [1]. There are currently no drugs that can reverse the development of OA and are only available to manage its symptoms of inflammation and pain [2]. Total hip arthroplasty, nicknamed operation of the century, has greatly improved the quality-of-life of patients especially when therapeutic measures have failed [3].

The development of Metal-on-Metal (MoM) and Metal-on-Polyethylene (MoP) hip arthroplasty marked an improvement in the range of motion and stability of the joint [4]. This proved to be particularly effective in younger patients especially those who practiced a more active life-style [5]. However, despite their success, the rate of adverse local tissue reactions (ALTRs) and adverse reactions to metal debris was observed with increased frequency [6,7]. ALTR/ARMDs are found in greater than 10% of patients with MoM hip implants and to a lesser extent with the more frequently used MoP Total hip arthroplasty implants [6]. ALTR/ARMDs can also occur in non-MoM total hip implants prostheses with CoCrMo with dual modular neck [7]. ALTR/ARMDs can manifest clinically as papillary/polypoid proliferation of the neo-synovial membrane (so-called pseudotumors) surrounding the implant site [7,8]. Detailed histological analysis has advanced the identification and description on the morphological features of ALTR/ARMDs as a specific diagnosis is associated with the types of inflammatory cells present in the reaction [7]. ALTR/ARMDs can be categorized as Type I – early onset and Type II – late onset [7]. Notwithstanding, clinically significant osteolysis has also been observed in late onset ALTR/ARMDs resulting in aseptic loosening of implant components [7].

The development of ALTR/ARMDs has been associated with elevated metal ions in the serum, although metallic particles including their size, shape and electric charge can be implicated [7,[9], [10], [11], [12], [13]]. The metal alloy used in MoM and MoP articulations is primarily composed of cobalt and chromium (CoCr). As corrosion and degeneration take place at the modular synovial membrane from joint capsule under microscopejunction and bearing surfaces of MOM prostheses, debris is released in local tissue and peripheral circulation due to the metallic wear, which results in the systematic elevation of Co and Cr concentrations [9]. In fact, a positive correlation has been reported between synovial fluid ion levels and those found in blood [9,14]. In several cohorts, joint fluid levels of Co and Cr ion levels have been reported as several orders of magnitude higher than that observed in blood fluid. Elevated serum levels of Co and Cr have been recorded in postoperative MOM implant patients after a follow-up of over 10 years [15]. The detrimental effects of elevated metal exposure have been linked to high failure rates of MOM prostheses [6,16].

The molecular mechanisms resulting in ALTRs are unclear. One theory suggest that metal ion release from the implants due to excessive bearing wear interacts with immune cells such as macrophages to generate a delayed hypersensitivity reaction leading to ALTR/ARMDs. Notwithstanding, an in vitro study demonstrated that Co and Cr ions function as haptens to act as anti-chemokines to suppress specific signaling pathways or act as immune response activators [17]. In addition, Co and Cr ions could also function as lymphotoxin factors inducing lymphocyte apoptosis [18]. However, lymphocyte activity is not consistently observed in ALTR/ARMDs.

The metal ion concentrations of Co and Cr are used to monitor the performance of hip implants. In the majority of centers, patients with blood concentrations of Co and Cr of 7 ​μg/L or greater are marked for surveillance for the possibility of developing ALTR/ARMDs; however, in cases of non-MoM implants with CoCrMo, lower blood concentrations of Co and Cr have been observed [7,10]. Given that implant fretting occurs within the synovial compartment, it is not surprising that the levels of metal concentration can reach upwards of 100-fold that found in the blood, particularly in patients with failed implants [[9], [10], [11], [12],^19^]. In one cohort, Co and Cr levels in the synovial fluid from patients with MoM hip implants ranged from 13.0 to 46433 ​μg/L (∼13 ​ppb–46 ​ppm) and 12.5–133120 ​μg/L (∼12 ​ppb–133 ​ppm), respectively [12]. It is currently unknown whether and how elevated metal ions present in the synovial fluid can directly effect the synovial membrane. In one study, using a cobalt-chromium-molybdenum alloy, it was found that there was a decrease in the proliferation and viability of fibroblasts, osteoblasts, macrophages by >50% at a dose of only 50 particles per cell [20].

The purpose of this study was to determine the prolonged effects of Co and Cr metal ions on synovial fibroblasts to better understand the potential impact on the synovial membrane.

## 2 Methods

### 2.1 Antibodies and materials

Cobalt (II) chloride (Cat. #C8661), chromium (III) chloride (Cat. #200050), and ascorbic acid (Cat. #A4544) were purchased from MilliporeSigma (Burlington, Massachusetts, USA). Antibodies anti-type I collagen (cat. #ab34710); anti-catalase (cat. #ab209211); anti-thioredoxin, anti-superoxide dismutase 1 (SOD1), and smooth muscle actin (cat. #ab179843) were purchased from Abcam (Cambridge, UK). Anti-phospho-Akt (Ser473) (Cat. #4060) was purchased from Cell Signaling Technology (Danvers, Massachusetts, USA).

### 2.2 Isolation of primary human synovial-like fibroblasts

Synovial membrane was acquired from three donors (ages 55–65; 2 males ​+ ​1 female) with informed consent undergoing total hip replacement. All procedures were approved by the institutional review board of the Jewish General Hospital. Samples of approximately 1 ​cm in size were collected by the orthopedic surgeon after surgery and transferred immediately in PBS containing 1% penicillin-streptomycin (Wisent Bio Products, Montreal, Quebec, Canada, cat. #450-200-EL) and 1% amphotericin B (cat. #450-105-QL). Synovium was sectioned with a scalpel into smaller pieces while soaking in PBS.

Sectioned synovial pieces were digested in 10 ​mL collagenase (MilliporeSigma Burlington, Massachusetts, USA; cat# C0130) [1 ​mg/mL] in Dulbecco's Modified Eagle Medium (DMEM) (Wisent Bio Products, Montreal, Quebec, Canada, cat. #319-006-CL) containing 1% penicillin-streptomycin +1% amphotericin B for 1 ​h at 37 °C rocking. Cell suspension was strained using 100 ​μm cell strainer followed by centrifuging at 400 rcf for 10 ​min. Pellet was washed in DMEM then resuspended in 10 ​mL culture medium (DMEM, 15% FBS (Wisent Bio Products, Montreal, Quebec, Canada, cat. #080–150), 1% penicillin-streptomycin and 50 ​μg/mL ascorbic acid) and added to a T-25 flask. Cells were cultured at 37 ​°C/5% CO_2_ and medium changed every 3 days. Cells were passaged six times before termination.

### 2.3 Alamar blue cell viability assay

Synovial fibroblast-like cells were cultured as pellets by centrifuging 3 ∗ 10^5^ ​cells in vented tubes at 400 rcf for 10 ​min. Pellets were cultured for 48 ​h prior prior to treatment. Pellets were maintained in DMEM containing 10% FBS and 1% penicillin-streptomycin supplemented with 10 ​μg/mL (10 ​ppm or approximately 42 ​μM) cobalt chloride (Co), 10 ​μg/mL (10 ​ppm or approximately 63 ​μM) chromium chloride (Cr) or both Co ​+ ​Cr (CoCr) and cultured for twelve days. Every three days including day 0, pellets were incubated with 500 μL medium containing 10% resazurin (alamarBlue reagent, ThermoFisher Scientific, Waltham, Massachusetts, USA, Cat. #DAL1025) for 3 ​h. Supernatant (100 ​μL) was added to a black 96-well plate and quantified for reductions in resazurin by measuring changes in its fluoresence (Ex 540 ​nm/Em 590 ​nm). Results were normalized to control pellet values incubated for the same amount of days. All experiments were performed in triplicate from three independent primary donor synovial fibroblasts. Pellets were rinsed in PBS prior to the addition of fresh medium supplemented with the indicated metal ions.

### 2.4 DNA content

DNA content was determined using Quant-iT dsDNA Assay kit (ThremoFisher Scientific, Waltham, Massachusetts, USA, Cat. #Q33120) following manufacturer's guidelines. Briefly, pellets (as prepared in the *Alamar Blue Cell Viability Assay* section) incubated for twelve days with culture medium supplemented with 10 ​μg/mL (10 ​ppm) cobalt chloride (Co), chromium chloride (Cr), both Co ​+ ​Cr (CoCr) or control (regular culture medium) were homogenized in 1 ​mL Tris-EDTA buffer. Sample (100 ​μL) was incubated with 100 ​μL of Picogreen Quant-iT dsDNA reagent and added to a black 96-well plate and measured using a spectrophotometer using Ex490/Em520 wavelengths. Quantification of DNA content was calculated using a standard curve from standards provided with the kit.

### 2.5 Live-Dead cell viability assay

Synovial fibroblast-like cells cell viability was performed as previously described [21]. Cells (5000 ​cells/well) were seeded on Lab-Tek chamber slides (ThermoFisher Scientific, Waltham, Massachusetts, USA; Cat. #155383) and incubated with medium supplemented with the indicated metal ions for twelve days. Cell were washed once with PBS and incubated with the LIVE/DEAD Cell Imaging Kit (ThermoFisher Scientific, Waltham, Massachusetts, USA; Cat. #L-3224) reagent for 15 ​min, and then observed by confocal microscopy (Zeiss LSM800 confocal laser-scanning microscope equipped with 488 and 543 ​nm laser lines). Images were captured using a 10× objective. The ratio of live cells (green) to total number of cells (green and red) was calculated using the cell counter function in ImageJ (National Institute of Health, USA) in four merged images per condition.

### 2.6 Cellular senescence

Synovial fibroblast-like cells were seeded in 6-well plates (1 ​× ​10^5^ ​cells/well) and cultured in medium supplemented with 10 ​μg/mL (10 ​ppm) cobalt chloride (Co), chromium chloride (Cr), both Co ​+ ​Cr (CoCr) or control (regular culture medium) for twelve days. Cells were washed in PBS and then processed using a Senescence Assay kit that measures senescent-associated beta-galactosidase activity (Abcam, Cat. #ab65351). Briefly, cells were fixed and incubated with a beta-galactosidase substrate for 24 ​h ​s at 37 ​°C. Cells were imaged using a Leica DM IL microscope. Blue presenting cells were indicative of senescence.

### 2.7 Hydrogen peroxide assay

Hydrogen peroxide detection in synovial fibroblast cells was performed using a Hydrogen Peroxide Assay kit following manufacturer's guideline (Abcam, Cat. #ab138874). Cells (5000/well) were seeded on Lab-Tek chamber slides (ThermoFisher Scientific, Cat. #ab138874) and incubated with medium supplemented with the indicated metal ions for twelve days. Cells were washed in PBS and incubated with a fluorescent peroxide indicator for 60 ​min at room temperature. Cells were then counterstained with DRAQ5 [1:2000] (ThermoFisher Scientific, Cat. #62251) prior to imaging by confocal microscopy using a Zeiss LSM800 confocal laser-scanning microscope equipped with 488 ​nm and 633 ​nm laser lines. Images were randomly collected throughout the well. Experiments on each donor cell line were performed in duplicate. Images were thresholded in ImageJ (National Institute of Health, USA) and positive cells were registered according to fluorescent intensity using the cell count function.

### 2.8 Western blotting

Western blotting was performed as previously described [22]. Briefly, synovial fibroblast-like cells were cultured in 6-well plates and incubated for twelve days or six days with the indicated metal ions. Cells were lysed in RIPA buffer containing protease inhibitor cocktail (MilliporeSigma, Burlington, Massachusetts, USA, Cat. #539131) and phosphatase inhibitor cocktail II (MilliporeSigma, Cat. #P5726). Cell extracts were centrifuged for 5 ​min at 10 ​000 ​rpm and lysate was quantified for protein content using Pierce BCA Protein Assay kit (ThremoFisher Scientific, Cat. #23225). Extracts were electrophoresed on 4–20% gradient gels (Bio-Rad) and transferred to PVDF membrane as previously described [22]. Blots were blocked in 5 % BSA in TBS and 0.1 % Tween 20 for 1 ​h and probed with anti-Collagen I [1:5000], anti-GAPDH [1:20000], anti-catalase [1:1000], anti-thioredoxin, anti-superoxide dismutase 1 (SOD1), and smooth muscle actin [1:1000], or anti-phospho-Akt (Ser473) [1:1000], in antibody solution (TBS, 1 % BSA and 0.1 % Tween 20) overnight at 4 ​°C. Blots were developed by incubation with anti-rabbit-HRP secondary antibodies [1:20000] and Amersham ECL Prime chemiluminescent detection reagent (GE Healthcare). Images were captured on a Molecular Imager VersaDoc (Bio-Rad). Densitometry was performed using ImageJ (National Institute of Health, USA). Density ratios were calculated using either GAPDH or actin where indicated.

### 2.9 Gene expression

Synovial fibroblast-like cells were cultured as pellets at a density of 3 ​× ​10^5^ ​cells/pellet and treated for six days in 0.5 ​mL medium supplemented with 10 ​μg/mL (10 ​ppm) of the indicated metal ions or control (culture medium alone). Total RNA was extracted using a total RNA mini-kit (Geneaid Biotech Ltd., New Taipei City, Taiwan) following manufacturer instructions. Complementary DNA was synthesised using a superscript Vilo cDNA synthesis kit (Thermo Fisher Scientific). Quantitative real-time PCR was quantified using an ABI 7500 fast light cycler using CYBR green master mix (Thermo Fisher Scientific) and specific primers (Table 1). Relative mRNA expression level was normalized to GAPDH using comparative CT analysis as previously described [23]. One-way ANOVA followed by Dunnett's multiple comparisons test was used to assess differences in gene expression. P ​≤ ​0.05 was deemed statistically significant.

**Table 1:** Primers for gene expression.

Genes	Primer sequence
COL1A1	F: 5′-GAGAGCATGACCGATGGATT-3’ R: 5′-CCTTCTTGAGGTTGCCAGTC-3’
NGF	F: 5′-TCAGCATTCCCTTGACACTG-3’ R: 5′-TGCTCCTGTGAGTCCTGTTG-3’
IL-1B	F: 5′-ACCTATCTTCTTCGACACATG-3’ R: 5′-ACCACTTGTTGCTCCATATCC-3’
TNFA	F: 5′-ACCACGCTCTTCTGCCTGCTG -3’ R: 5′-TACAACATGGGCTACAGGCTT -3’
GAPDH	F: 5′-GCTCTCCAGAACATCATCCCTGCC-3’ R: 5′-CGTTGTCATACCAGGAAATGAGCTT-3’

### 2.10 Statistical analysis

Data were analyzed by Two-way ANOVA followed by posthoc Dunnett's or Tukey's multiple comparisons' tests. A p-value of less than 0.05 was considered statistically significant. Graphs and statistical analysis were performed using GraphPad Prism version 8.3.0 software (GraphPad Software, San Diego, California USA).

## 3 Results

### 3.1 Cell growth, viability, and senescence following Co and Cr exposure

**Fig. 1:**
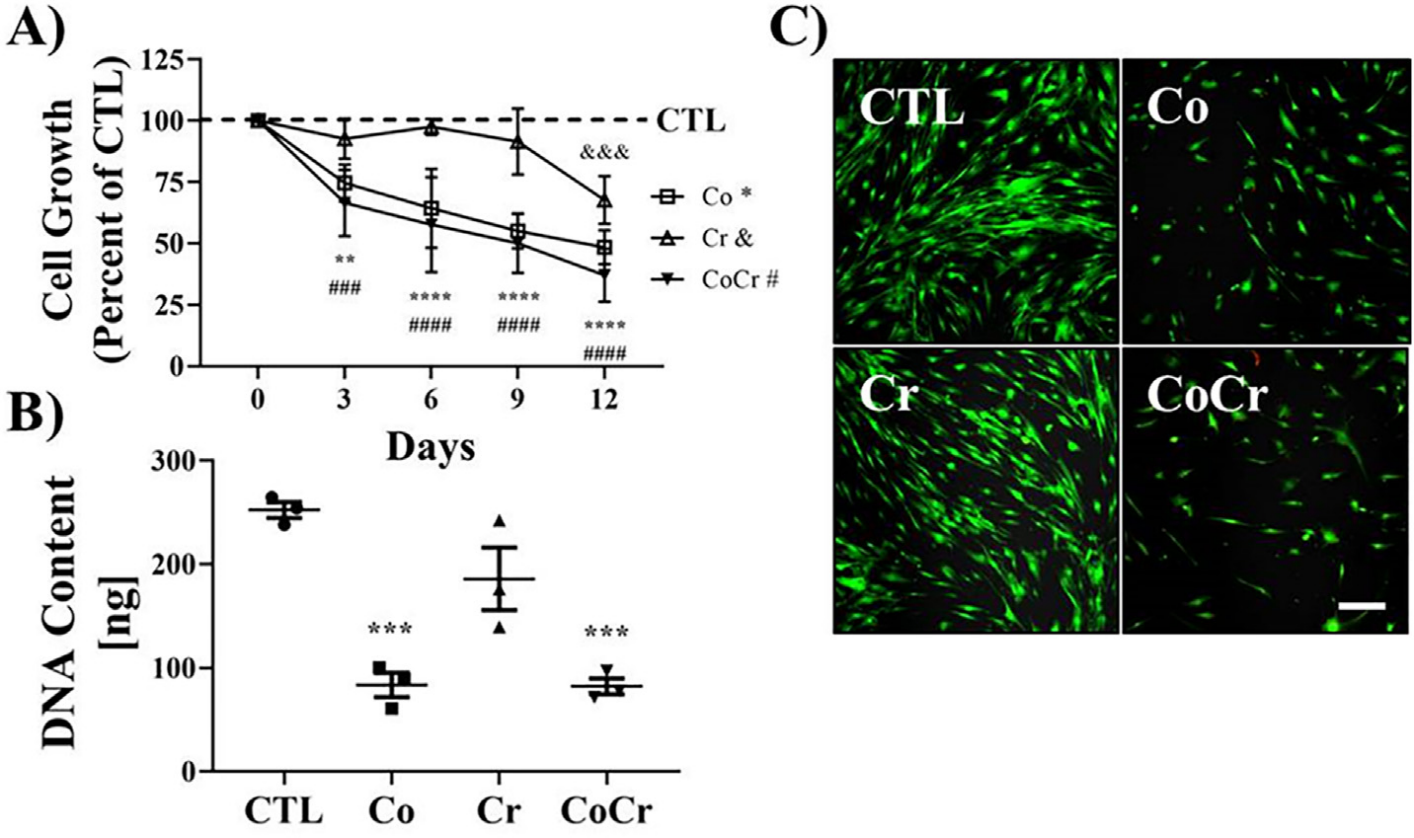
**Effect of CoCr on human synovial fibroblast viability**. Synovial fibroblasts (SFs) were treated with medium supplemented with 10 ​ppm cobalt (Co), chromium (Cr), both (CoCr), or untreated (CTL). **A)** SFs from three donors were cultured as pellets and incubated in regular growth medium (CTL) or medium supplemented with the cobalt (Co), chromium (Cr) or cobalt ​+ ​chromium (CoCr) for a total of 12 days. Every 3 days beginning with Day 0 pellets were incubated with Alamar blue reagent and compared to control pellets on the same day. Means ​± ​SDs; Two-Way ANOVA posthoc Tukey's multiple comparison test; ∗∗, p ​< ​0.01; ###, &&&, p ​< ​0.001; ∗∗∗∗, ####, p ​< ​0.0001; n ​= ​3 donors. **B)** DNA content from cells cultured as pellets following 12 days incubation in medium supplemented with the indicated metals. Means ​± ​SEMs; ANOVA posthoc Tukey's multiple comparison test, ∗∗∗, p ​< ​0.001, n ​= ​3. **C)** SF cells cultured in chamber slides and treated with the indicated metals for 12 days and assayed for cell viability using a fluorescent Live-Dead viability assay and imaged by confocal microscopy. Scale bar ​= ​100 ​μm.

Synovial fibroblast-like cells (SFs) from three independent donors were exposed to 10 ​μg/mL (10 ​ppm) Co, Cr or both Co and Cr to determine any effects on cell growth. As shown in Fig. 1A, Co significantly decreased the growth curve of SFs when measured using the Alamar blue cell viability assay. Unlike Co, Cr did not significantly affect cell growth until after nine days of exposure. This was not the case when Cr was co-incubated with Co, the effects on SF growth were similar to cells exposed to Co alone. DNA content was measured to determine total cellular content following twelve days of metal ions exposure. Similar to the results presented in Fig. 1A, when cells were exposed to Co, alone or in combination with Cr, DNA content was significantly decreased by approximately half that of controls (Fig. 1B). Cell viability was also measured using a Live-Dead assay to determine whether Co or Cr induced SF cell death. As shown in Fig. 1C, Co alone or in combination with Cr decreased the presence of viable cells (green). We did not observe any increases in the number of dead cells following Co exposure (red).

**Fig. 2:**
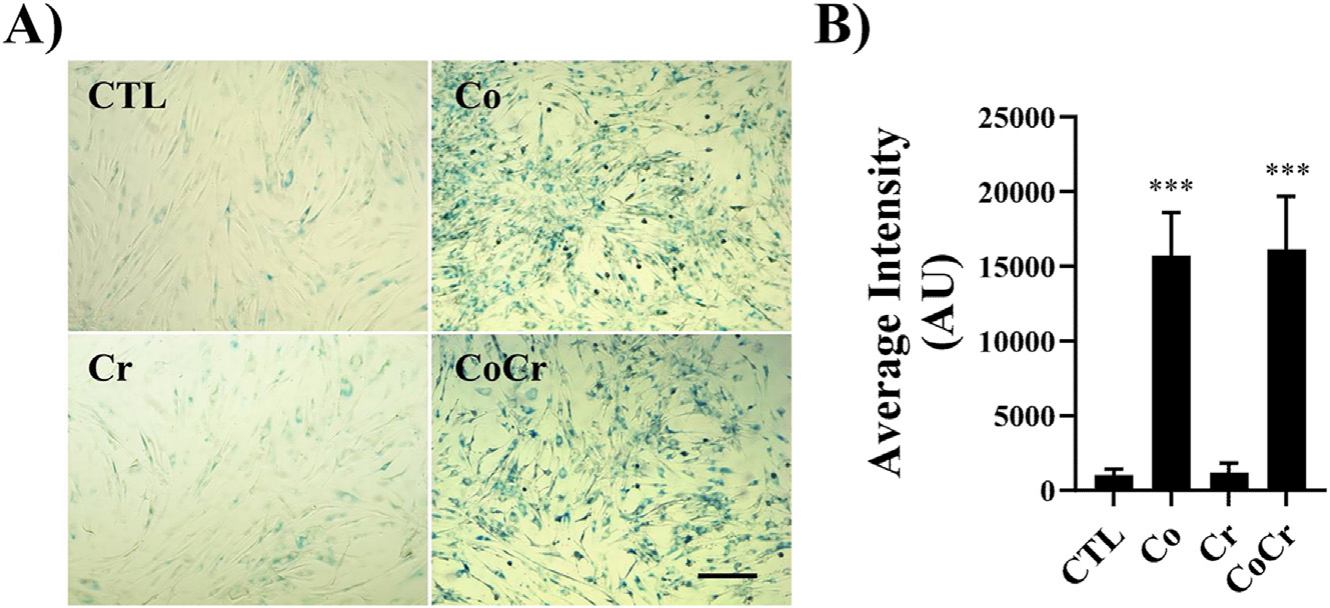
**Cellular senescence induced by CoCr ions**. Synovial fibroblasts were cultured for twelve days in medium supplemented with the indicated metal ions. **A)** Cells were fixed and stained for senescence-associated beta-galactosidase activity (blue staining). Representative images from a donor cell line. Scale bar ​= ​50 ​μm. **B)** Images were analyzed by ImageJ for blue staining. Means ​± ​SEMs; ANOVA posthoc Dunnett's multiple comparison test, ∗∗∗, p ​< ​0.001, n ​= ​3.

Since we did not observe increases in cell death in SF cells following Co treatment, we investigated the possibility that Co may be inducing cellular senescence. As shown in Fig. 2a, when SF cells were exposed to 10 ​ppm of either Co or Co and Cr together for twelve days, there was a striking appearance of senescent cells as determined when measured for beta-galactosidase activity (blue staining). When images were analyzed for intensity of blue staining, there were significant increases in the Co and Co and Cr treated cells (Fig. 2b). This was not the case in control SF cells or with cells exposed to Cr alone.

### 3.2 Oxidative stress and hydrogen peroxide accumulation in Co and Cr exposed cells

**Fig. 3:**
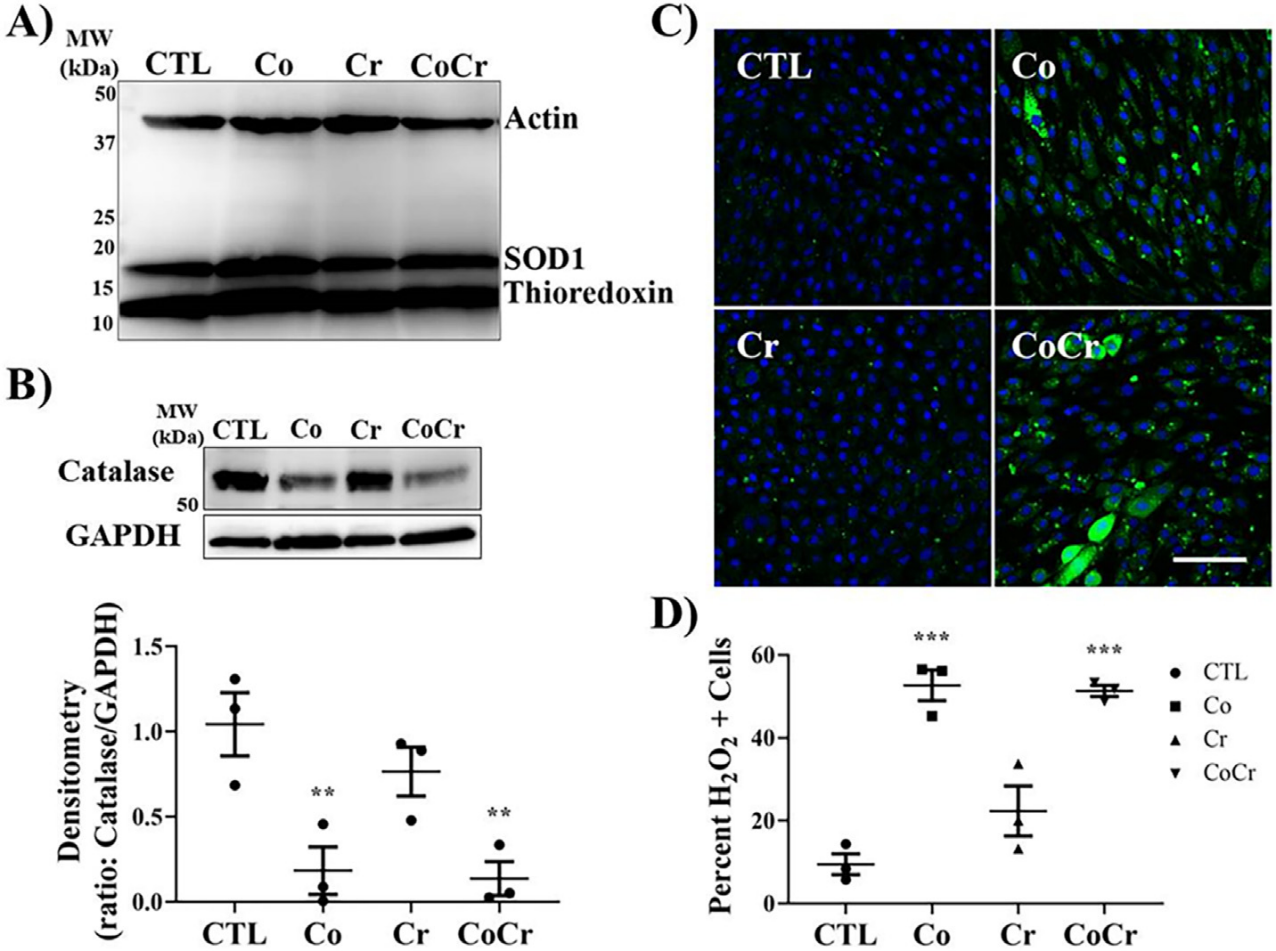
**Cobalt ions downregulate catalase and increase the accumulation of hydrogen peroxide in synovial fibroblasts**. **A)** Western blot for the expression of the oxidative stress enzymes: SOD1 and Thioredoxin, and actin. **B)** Western blot for the expression of catalase and GAPDH. Densitometry on blots presented in **B)**. Dots represent individual donor cells, means ​± ​SEMs; ANOVA posthoc Dunnett's using CTL as comparison. **C)** SFs cultured in chamber slides were incubated for 12 days with the indicated metals and imaged using a hydrogen peroxide assay (Abcam) by confocal microscopy. Green cells are positive for H2O2 accumulation. Cells were counterstained with DRAQ5 to visualize the nucleus (blue). **D)** Percent of cells demonstrating H_2_O_2_ accumulation. Dots represent individual donor cells, means ​± ​SEMs; ANOVA posthoc Dunnett's using CTL for comparison.

Oxidative stress in cells is controlled by several factors. There are three principle enzymes that regulate this process, thioredoxin, superoxide dismutase type 1 (SOD1), and catalase. To determine if Co or Cr had any effect on the expression of oxidative stress enzymes we ran Western blots on lysates from SF cells. Following twelve days of metal ion exposure, we did not observe any differences in the expression of SOD1 or thioredoxin in SF cells (Fig. 3A). There was, however, significant changes in the expression of catalase, enzyme involved in the decomposision of hydrogen peroxide (H_2_O_2_), when SF cells were exposed to with Co or Co ​+ ​Cr (Fig. 3B). Treatment of SF cells with Cr did not significantly alter the expression of catalase when compared to controls.

Downregulation in the expression of catalase may result in increases in H_2_O_2_ cellular content. To determine if downregulation of catalase by Co affected the generation H_2_O_2_, we probed the cellular content of H_2_O_2_ using a hydrogen peroxide fluorescent assay. As shown in Fig. 3C and D, there were significant increases in the number of cells that were positive for H_2_O_2_ accumulation following twelve days of treatment with either Co or Co ​+ ​Cr. There were no significant differences in the number of H_2_O_2_ positive cells (H_2_O_2_+) when treatment was with Cr alone.

### 3.3 Dysregulation of collagen synthesis and Akt activity in Co-exposed SF cells

**Fig. 4:**
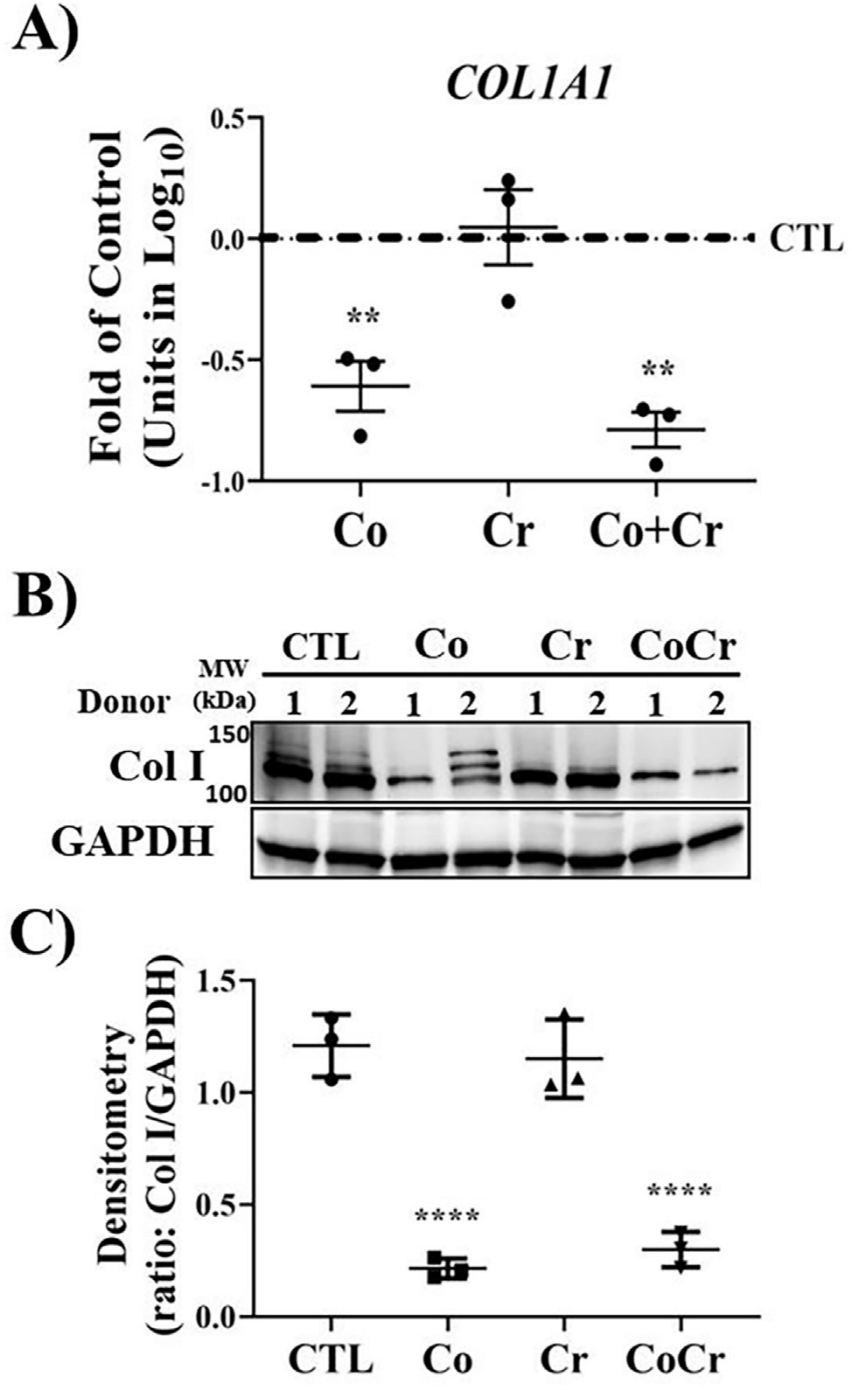
**Effect of CoCr on collagen synthesis**. **A)** Fold change in gene expression for collagen type 1 (Col I) in synovial fibroblasts exposed to cobalt (Co), chromium (Cr) or CoCr for 6 days when compared to controls (CTL). Means ± SEMs; ANOVA posthoc Dunnett's; ∗, p ​< ​0.05; n ​= ​3 individual donors. **B)** Western blot for the expression of collagen type I (Col I) in SF cells following 12 day exposure to the indicated metal ions. **C)** Densitometry on blots presented in **B)**. Means ± SEMs; ANOVA posthoc Dunnett's; ∗, p ​< ​0.05; n ​= ​3 individual donors.

The primary matrix protein constituting that synovial membrane is collagen. SFs are the principle cell type regulating collagen content in the synovium. To determine if Co or Cr altered the expression of collagen in SFs, we preformed RT-qPCR on cells incubated with the indicated metal ions for six days. As shown in Fig. 4A, the expression of collagen type I (Col I) was significantly reduced following Co treatment alone or in combination with Cr. Changes in Col I protein synthesis was verified by Western blotting after twelve days of metal ion exposure in SF cells. Similar its effects on mRNA expression, Co exposure significantly reduced the synthesis of Col I either alone or in combination with Cr (Fig. 4B and C).

**Fig. 5:**
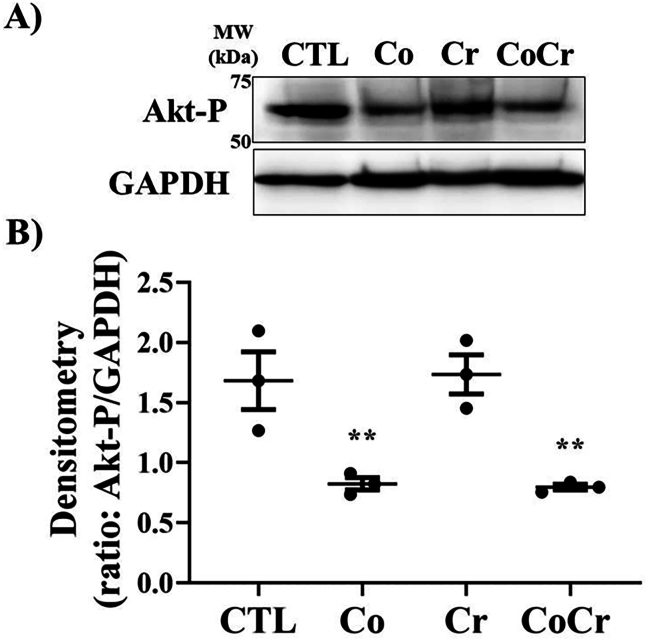
**Effects of cobalt on Akt activity in synovial fibroblasts**. **A**) Western blots for Akt phosphorylation (Ser 473) in synovial-like fibroblasts following six days in medium supplemented with the indicated metal ions. **B**) Densitometry on blots presented in A). GAPDH was used as a loading control. Means ± SEMs; ANOVA posthoc Dunnett's; ∗, p ​< ​0.05; n ​= ​3 individual donors.

The phosphatidylinositol-3-kinase/Akt (PI3K/Akt) pathway is known to regulate proliferation and matrix protein synthesis in fibroblasts [24,25]. To determine if Co or Cr can modulate this pathway we performed Western blotting on the phosphorylated active form of Akt. As shown in Fig. 5, following six days of exposure to medium supplemented with Co, the activity of Akt was decreased by approximately half that of control cells. SF cells treated with Cr alone did not significantly alter the phosphorylation status of Akt, however, combination of Cr with Co did decrease its activity to levels observed when Co was applied separately (Fig. 5A and B).

### 3.4 Oxidative stress and upregulation of markers for inflammation and pain

**Fig. 6:**
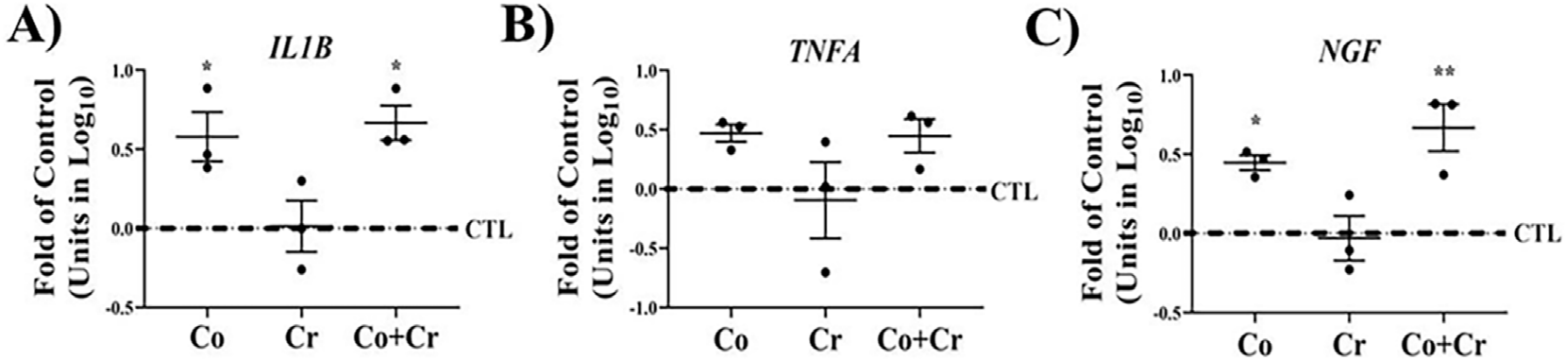
**Effect of CoCr on markers of inflammation and pain**. Gene expression for **A)** interleukin 1β, **B)** TNF-α, and **C)** NGF in synovial fibroblasts with the indicated metal ions for 6 days. Means ± SEMs; ANOVA posthoc Dunnett's; ∗, p ​< ​0.05; n ​= ​3.

Oxidative stress has been associated with aberrant cellular activity. To determine if Co or Cr affected other aspects of SF cellular activity, we measured changes in the expression of markers of inflammation and pain. As shown in Fig. 6, Co significantly increased the expression of IL-1β and NGF in SF cells. A similar pattern was observed in cells incubated with both Co and Cr. Although the effect of Co or Co and Cr on TNFα expression was not significantly different from control cells, there was a trend towards its upregulation (Fig. 6B). We did not observe any significant changes in the expression of IL-1β, NGF or TNFα in SF cells exposed to Cr alone.

## 4 Discussion

Elevated levels of metal ions Co and Cr in the serum, urine and synovial fluid of patients with MoM and MoP implants has been shown to associate with the development of ALTR/ARMDs [7,8]. Although patients with serum levels of Cr and Co of >7 ​ppb (7 ​μg/L) are considered at risk of developing ALTR/ARMDs, concentrations in the synovial fluid have been shown to exceed 100-fold that found in serum [[9], [10], [11], [12],^26^]. In several cohorts, median levels of Co and Cr ions in joint fluid were approximately 1000 ​μg/L (1 ​ppm) and upwards of 10000 ​μg/L (10 ​ppm) in failed implants [9,12]. Notwithstanding, Co and Cr ion were estimated at concentrations of over an order of magnitude higher in synovial tissue when compared to the surrounding synovial fluid [27].

ALTR/ARMDs are described radiologically as cystic or solid masses and histologically as papillary/polypoid ne-synovial proliferations with loss of lining cell layer, predominantly lymphocytic or macrophage infiltrate with or without multinucleated giant cells and tissue necrosis [7,8,28]. ALTR/ARMDs can be destructive, drastically limiting the range of motion the affected joint, and in many instances lead to pain in the hip region [8]. Although the etiology of ALTR/ARMDs is unclear, several studies have demonstrated histological evidence indicating that it may be a product of a delayed hypersensitivity immune response [8,28,29]. For instance, histological examination of periprosthetic soft tissue from failed MoM implants have shown a lymphocytic infiltrate predominantly composed of T-cells lymphocytes associated with particle-laden macrophages and a variable amount of neo-synovial stromal proliferation. Once activated, T lymphocytes release cytokines that can induce the formation of giant macrophages, incite cell death, and trigger the proliferation of fibroblasts [8]. In addition, Co and Cr ions and debris have demonstrated direct effects on macrophages [8,30]. Increasing concentrations of chromium enhanced the release of pro-inflammatory cytokines TNFα and IL6 in peripheral blood mononuclear cells [31]. In one study, high concentrations of both CoCr were cytotoxic to PBMCs, however, unlike chromium where apoptosis was the main outcome, cobalt induced necrosis [32]. It was also determined that high concentrations of both CoCr can induce necrosis in cultured macrophages in a time-dependent manner [33,34]. Whether Co and Cr ions can directly alter the physiology of the synovial membrane and whether this can play a role in ALTRs remains unknown.

In the present study, the effect of Co and Cr ions on human SF cells was investigated. We provide evidence that in the early stages of metal ion exposure, synovial-like fibroblasts undergo growth arrest and cellular senescence due to increased oxidative stress imparted by cobalt exposure. Concentrations of Co and Cr used were within the upper limits of levels previously measured in the synovial joint of patients with MoM and MoP hip implants. When SF cells were cultured in medium supplemented with Co, significant changes in cell growth were observed following 12 days of exposure. Similar changes in SF cell growth were apparent as early as 72 ​h when cells were treated with cobalt. However, unlike the case with Cr, cells exposed to Co alone demonstrated a modest decrease in cell growth at the end of 12 the day incubation period. The rate of cell growth inhibition following combination of Co and Cr was similar to exposure of Co alone, suggesting Cr did not induce a synergistic affect on cell proliferation. Interestingly, neither Co nor Cr increased cell death as indicated by a Live/Dead assay. Similar results were demonstrated in the study by Eltit et al. where Co induced a down regulation of genes associated with cell cycle growth in synovial fibroblasts [26].

Since neither metal appeared to be inducing cell death despite having a significant impact on cell growth, we sought the possibility that SF cells were undergoing cellular senescence. Indeed, a beta-galactosidase activity assay confirmed the presence of cell senescence following exposure of SF cells to Co. Similar results were obtained when cells were treated with Co and Cr; however, exposure to Cr alone did not result in any appreciable changes in cellular senescence. Cellular senescence is known to play a role in normal physiology particularly in wound healing, embryogenesis and ageing, however, when prolonged, senescence can result in pathological conditions such as chronic inflammation, decreased immunity, tumour development, chronic kidney disease and osteoporosis [35]. Although senescent cells are typically incapable of proliferating, they are not dormant and can actively secrete factors such as proinflammatory cytokines termed the senescence-associated secretory phenotype. Indeed, Co-induced senescence of SF cells resulted in significant increases in the expression of IL-1β and the neurotrophic factor NGF. Significant changes in neurotrophic and inflammatory factors were not observed when cells were exposed to Cr alone. Several studies have demonstrated elevated levels of cytokines in failed implants including the proinflammatory cytokines IL-1, IL-6 and TNFα [26,36]. In the study by Kolatat et al. where they investigated the cytokine profiling of failed MoM and dual modular neck total hip replacements, it was discovered that in addition to increased levels of IL-6 and IL-8, there were also elevations in INFγ and its associated chemokines MIG and IP-10 [37]. Here, in addition to proinflammatory cytokines, we observed increases in the expression of NGF. Cobalt-induced upregulation of NGF in SF cells could be one of the factors implicated in the development of painful ALTRs.

Several metals are known to undergo redox recycling reactions in cells generating elevating levels of intracellular reactive oxygen species (ROS) such as superoxide anions, hydroxyl radicals and hydrogen peroxide and nitric oxide [38]. As ROS accumulates, cells can be subjected to various unwarranted effects and without adequate capability of neutralizing ROS, lipid peroxidation, DNA damage and protein modifications can ensue. Notwithstanding, ROS has also been shown to directly or indirectly alter the activation of signaling pathways and transcription factors implicated in regulating or neutralizing intracellular ROS in a cell-type dependent manner. In the study by Scharf et al., both Co and Cr ions increased the generation of ROS and carbonylated a large fraction of intracellular proteins (an indicator of oxidative damage) in macrophages [39]. Prolonged exposure to ROS is a significant driver of cellular senescence [35]. The phospoinositide 3-OH kinase (PI 3-kinase)/Akt pathway is an important signaling pathway providing cellular growth and survival largely through inhibition of the apoptotic machinery [25]. ROS has been shown to directly regulate Akt activity, for instance basal levels of cellular H_2_O_2_ has been shown to induce the phosphorylation of Akt providing protection of cells from damage mediated by oxidative stress [25]. However, dephosphorylation and inactivation of Akt ensues when H_2_O_2_ levels become excessive [40]. Interestingly, we observed significant decreases in the phosphorylation of Akt when cells were incubated with Co for a prolonged period. This suggested that H_2_O_2_ may be elevated in cells exposed to Co. Indeed, a significant number of cells demonstrated elevated levels of H_2_O_2_ when exposed to Co and not Cr. A similar effect was also observed in the study by Salloum et al. where they demonstrate that Co and not Cr in a dose-dependent manner reduces cell viability and increases oxidative stress in macrophages [41].

The redox cycling of many metals typically results in the generation of superoxide anion radicals [38]. The key cellular enzyme responsible for regulating superoxide levels is Superoxide Dismutase (SOD1) [42]. SOD1 catalyzes the conversion of superoxide radicals to oxygen and H_2_O_2_ [43]. Results from our study revealed that neither Co or Cr had any effect on the expression SOD1 enzyme in SF cells. Interestingly, the expression of Theoredoxin-1, an enzyme that also regulates cellular redox balance through the reversible thiol-disulfide exchange reaction [44], was unchanged following Co and/or Cr exposure. Scavenging of H_2_O_2_ occurs through a variety of factors such as glutathione peroxidase and peroxiredoxins, however, one prominent factor in the decomposition of H_2_O_2_ is catalase [45]. Catalase is expressed primarily in peroxisomes and to some extent the cytosol [46]. Oxidative stress tolerance including longevity have been attributed to the expression of catalase [47]. When SF cells were exposed Co, the expression of catalase was significantly reduced. This was contrary to the exposure of SF cells to Cr, cellular catalase levels remained similar to controls. Although catalase expression was decreased in SF cells following exposure to co-treatment with Co and Cr, the levels were similar to Co treatment alone, suggesting the combined treatment did not impart a synergistic effect. Interestingly, in a previous report using murine macrophages, Cr and not Co was able to directly inhibit the enzymatic activity of catalase; however, the concentrations required to achieve considerable inhibition of the enzyme was 400 ​μM or more (>100 ​ppm), higher than what we used in our study and greater than what is typically measured in patient samples [39]. Given the importance of catalase in the decomposition of H_2_O_2_, it is possible that changes in its expression through Co exposure could explain the cellular accumulation of H_2_O_2_. In an earlier report, Co was demonstrated to effect the synthesis and degradation of hepatic catalase *in vivo* [48]. Notwithstanding, arsenic was shown to decrease the expression activity and synthesis of catalase in a dose-dependent manner [49]. It is interesting to note that transient exposure of certain cell types to H_2_O_2_ results in phosphorylation and activation of Akt leading to the decrease in catalase expression. This has been demonstrated to occur through the inhibition of the transcription factor FoxO [50]. FoxO has been shown to positively regulate catalase expression by interaction with its promotor [51]. This is contrary to the effects of prolonged exposure of SF cells to Co where we observed decreases in Akt activation and the increased presence of cellular H_2_O_2_. Whether Co has a direct effect on FoxO transcription factors remains unknown. In addition, it is possible that Co may be regulating other transcription factors implicating in catalase expression [52]. The exact mechanism for the effect of Co on catalase regulation is unclear.

Some limitations to the study include whether impairment in the homeostasis of the synovial membrane may be a primary step toward the development of adverse reactions in metal ion toxicity. Co may have induced SF cells to become senescent and upregulate inflammatory and pain markers typified of a senescence-associated secretory phenotype [53]. The results in this study may reflect the early events associated with SF cells to Co exposure. Indeed, ALTRs are fibrous and inflamed tissues resulting from chronic exposure to metal ions. It is possible that during the initial stages of metal ion exposure, senescent SF cells recruit macrophages, leading to a remodeling of the local synovial microenvironment, driving inflammation and the invasion of activated extracellular matrix fibroblasts. It remains to be determined whether this phenotype is consistent *in vivo*. It also remains unclear whether there are any sex-related differences following exposure of SF cells to Co.

It is important to note that the detrimental effects of elevated metal exposure are not limited to the development of pseudotumors but include endocrine, cardiac and neurological disorders and potentially harmful effects on the liver and kidneys [54]. Antioxidants and radical scavengers have been shown to be chemoprotective against metal toxicity [38]. For instance, the antioxidant *N*-acetylcycteine (NAC) has been demonstrated to block the toxic effects of several metals including cobalt, chromium and cadmium [55]. Perhaps patients at risk of developing ALTRs due to elevated levels of Co and Cr can be prescribed NAC, although studies are needed to determine its benefit.

## 5 Conclusion

**Fig. 7:**
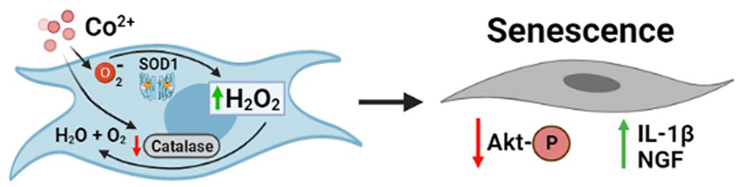
**Schematic representing a mechanism for the effect of cobalt on synovial fibroblasts.** Schematic was produced using https://biorender.com.

In conclusion, we provide evidence that elevated levels of Co and not Cr ions may adversely affect the neo-synovial membrane by inducing a senescent associated secretory phenotype in synovial fibroblasts that could contribute to the development of ALTR/ARMDs ​(see Fig. 7). MoM and MoP implants with CoCrMo/Ti metallic junctions are still in use worldwide, and a large number of patients who carry these implants are monitored for changes in their metal ion levels and occurrence of adverse events. Understanding the role of cellular reactions in the neo-synovial membrane in the development of ALTR/ARMDs may provide insights useful for safer implant designs in the future.

## Author contributions

MPG, JA and FM designed the study and experiments. RA, MOD, MA, OLH and SGB conducted sample collection and isolation of synovial fibroblasts. MPG, RA, MOD and MA conducted experiments. MPG, RA and LME performed analysis of the data. MPG, HJIS, JA and FM wrote the manuscript.

## Role of the funding source

This work was supported by the Canadian Institutes of Health Research (CIHR).

## Declaration of competing interest

The authors declare that they have no known competing financial interests or personal relationships that could have appeared to influence the work reported in this paper.
